# Psychometric properties of the Childhood Trauma Questionnaire–Short Form (CTQ-SF) in adolescents from the Peruvian Amazon

**DOI:** 10.3389/fpsyg.2026.1939936

**Published:** 2026-10-08

**Authors:** Marita Mily Paz-Vilchez, Yosmeli Moreno-Acosta, Julio Cjuno, Jhordan Mayta-Llanos

**Affiliations:** 1 Postgraduate Unit of Psychology, Universidad Peruana Unión, Lima, Peru; 2 Professional School of Psychology, Universidad Peruana Unión, Tarapoto, Peru

**Keywords:** adolescents, childhood trauma, confirmatory factor analysis, construct validity, factorial structure, internal consistency, psychometric validation

## Abstract

Childhood trauma is a significant public health problem owing to its effects on the socioemotional development of adolescents, which calls for validated assessment instruments. This study aimed to adapt and examine the psychometric properties of the Childhood Trauma Questionnaire–Short Form (CTQ-SF) in adolescents from the Peruvian Amazon. An instrumental, cross-sectional study was conducted with 878 adolescents aged 12–17 years (M = 14.0; SD = 1.46) from public secondary schools in the San Martín Region, Peru, randomly divided into two subsamples of 439 participants each for exploratory (EFA) and confirmatory factor analysis (CFA); reliability, convergent and discriminant validity, and measurement invariance across sex were also examined. The original five-factor model did not show adequate performance. After removing items with weak loadings or redundant content, a three-factor, 18-item structure emerged—lack of perceived family support/neglect, sexual abuse, and physical–emotional abuse—which showed adequate fit (CFI = 0.958; TLI = 0.951; RMSEA = 0.051; SRMR = 0.067) and satisfactory internal consistency (ordinal *α* and *ω* = 0.76–0.95). Convergent validity was adequate for sexual abuse and physical–emotional abuse (AVE = 0.81 and 0.50) and borderline for lack of perceived family support/neglect (AVE = 0.42), whereas discriminant validity was supported for all factor pairs (HTMT = 0.262–0.626). Full scalar invariance across sex was also established (ΔCFI ≤ 0.002; ΔRMSEA ≤ 0.003). These findings indicate that the Peruvian CTQ-SF is better represented by a three-factor correlated structure, yielding a brief version that allows comparisons between sexes; nevertheless, because its temporal stability and external validity remain to be examined, its use should currently be restricted to research and preliminary screening purposes.

## 1 Introduction

Childhood trauma is an alarming problem worldwide that may lead, in adulthood, to mental health complications such as difficulty coping with ordinary stress and the onset of disorders or mental illnesses including anxiety, post-traumatic stress, depression, eating disorders, and in some cases suicide (Segovia-Monga et al., 2022).

It has been reported that 36.3% of children experience trauma; the World Health Organization (WHO) defines trauma as events experienced in childhood in which psychological and physical rights are violated (World Health Organization, 2024). Nearly a quarter of adolescent girls aged 15–19 worldwide (approximately 70 million) reported having experienced some form of physical violence since the age of 15 (UNICEF, 2014). In Peru, this problem has become more visible through social media, everyday conversations, and scientific research, which show that children and adolescents are the population most vulnerable to family neglect (Lazo et al., 2017). Statistics indicate that 26% of adolescents were affected by emotional violence, 22.8% by physical violence, and 66.3% by sexual violence (Instituto Nacional de Estadística e Informática, 2019).

These traumatic experiences during childhood, as well as exposure to violence, represent significant risk factors in adolescence and adulthood (Reyes-Gaspar and Charry-Mendez, 2023). Individuals who experienced greater adversity during childhood are prone to more intense feelings of loneliness and a less optimistic outlook during adolescence (De La Cruz-Valdiviano et al., 2025). Children who have suffered physical abuse, sexual abuse, and neglect live with trauma and have difficulties relating to those around them (Huamaní et al., 2022).

The factorial structure of the CTQ-SF, however, has not proven invariant across populations. Recent meta-analytic evidence shows that its original five-factor model exhibits inconsistent replicability and that the physical neglect subscale recurrently displays the lowest internal consistency, which has led to proposing alternative solutions with four or fewer factors across different cultural contexts (Badenes-Ribera et al., 2024; Peng et al., 2023). These structural variations are especially relevant when adapting the instrument to adolescent populations in non-English-speaking countries, where cultural differences in the conceptualization of care and neglect may alter how items empirically cluster (Hoeboer et al., 2026).

The CTQ-SF, which measures a history of childhood abuse and neglect, was created and validated in English in a United States sample. The questionnaire comprises 28 items, of which 25 are distributed across five dimensions (emotional abuse, physical abuse, sexual abuse, emotional neglect, and physical neglect) and 3 are validity items (Bernstein et al., 2003). It has been used in clinical samples from several countries, such as Hungary (Kenézlői et al., 2023), Korea (Kim et al., 2013), Nigeria (Aloba et al., 2020), the Netherlands (Thombs et al., 2009), Slovakia (Petrikova et al., 2021), and Danish (Kongerslev et al., 2019), where it maintained good reliability and validity indices. However, in China the physical and emotional neglect subscales merged into a single neglect subscale, leaving the questionnaire with four factors (Kim et al., 2013; Peng et al., 2023), and in Chile a five-factor structure with 23 items was re-evaluated, removing items 1 and 6 from the physical neglect scale (Behn et al., 2020).

In Peru, no studies have been found providing information on the psychometric properties of this instrument in adolescent populations, despite the prevalence of abuse and neglect in this group. The aim of this study is to adapt the Childhood Trauma Questionnaire–Short Form (CTQ-SF) in adolescents from public secondary schools in the San Martín Region, Peru and to determine the content validity, reliability, and factorial structure of the instrument. This gap is problematic because applying the original five-factor structure without local validation may yield unreliable scores and misinform screening and research decisions in this population.

## 2 Materials and methods

### 2.1 Design and setting

This is a quantitative, non-experimental, psychometric study (Ato et al., 2013).

### 2.2 Participants

The population comprised Peruvian adolescents of both sexes from the San Martín Region, Peru. Participants were recruited from two public secondary schools in the region: one located in the district of Rioja (483 students) and one in the district of El Dorado (472 students), yielding a total of 955 invited students. A non-probability convenience sampling method was used. Prior to data collection, during an earlier visit to the schools, informed consent forms were distributed to parents or guardians. Of the invited students, 46 (4.8%) did not return the signed consent form and were therefore not assessed, resulting in 909 adolescents who completed the questionnaire. Subsequently, 6 participants were excluded for being outside the established age range and 25 for incomplete data, yielding a final sample of 878 adolescents. See Figure 1.

**Figure 1:**
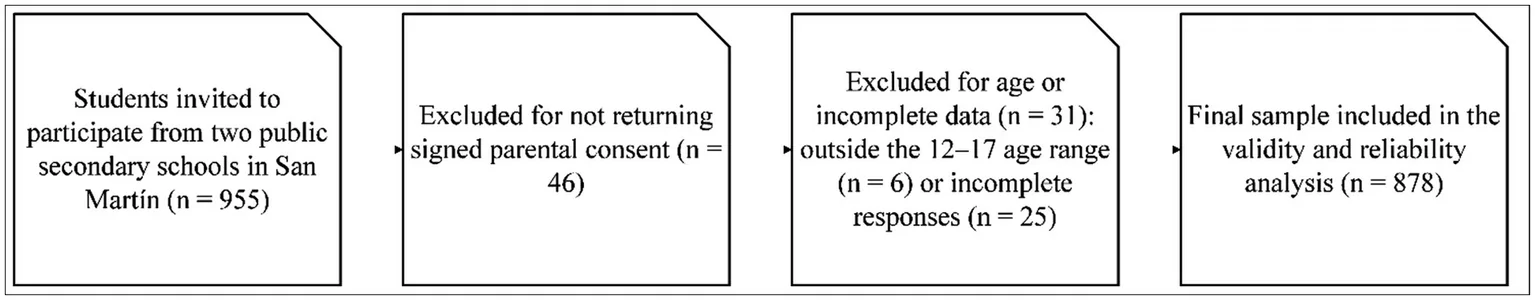
Participant flowchart.

The inclusion criteria were as follows: being between 12 and 17 years of age, being of either sex, having informed parental or guardian consent to participate, providing informed assent, and holding Peruvian nationality. The exclusion criteria were: presenting any temporary or permanent physical or mental condition that precluded a coherent response; leaving items unanswered; and selecting the same response option for all items (invariant response pattern). Finally, the sample of 878 adolescents was randomly divided into two subsamples: 439 participants for the exploratory factor analysis (EFA) and 439 for the confirmatory factor analysis (CFA).

To estimate the minimum sample size required for the CFA of the final three-factor, 18-item model (df = 132), a sample-size adequacy and power assessment for the final CFA model based on the RMSEA close-fit test was conducted, specifying *α* = 0.05, statistical power (1 − *β*) = 0.80, a null RMSEA of 0.05, and an alternative RMSEA of 0.08. Under these conditions, the minimum required sample size was 110 participants. The subsample used for the CFA (*n* = 439) therefore exceeded this threshold and provided statistical power above 0.99 to detect model misfit. This subsample also yielded a participant-to-item ratio of approximately 24:1, well above conventional recommendations for factor analysis (MacCallum et al., 1996).

### 2.3 Instrument

The instrument adapted to the Peruvian context is the Childhood Trauma Questionnaire–Short Form (CTQ-SF), developed by Bernstein et al., (2003) and composed of 28 items, of which 25 form 5 factors (EA = emotional abuse; PA = physical abuse; SA = sexual abuse; EN = emotional neglect; PN = physical neglect) and 3 are validity items, with a Likert-type response format (never, rarely, sometimes, often, very often), such that, for the subscales composed of five items, scores range from 5 to 25 points. In an adolescent subsample it demonstrated good criterion validity and reliability, with Cronbach’s alpha of 0.89 for Emotional Abuse, 0.89 for Emotional Neglect, 0.95 for Sexual Abuse, 0.86 for Physical Abuse, and 0.78 for Physical Neglect. Nevertheless, given that the original five-subscale structure was not replicated in the present sample, scoring is derived from the empirical three-factor solution identified in this study. Scores are obtained by summing the items of each factor, yielding possible ranges of 7–35 for lack of perceived family support/neglect, 5–25 for sexual abuse, and 6–30 for physical–emotional abuse (Bernstein et al., 2003).

### 2.4 Cultural adaptation procedure

The cultural adaptation of the CTQ-SF into Peruvian Spanish was carried out following international recommendations for the translation and cross-cultural adaptation of self-report instruments (Beaton et al., 2000; Hernández et al., 2020), and comprised three stages.

Stage 1. Forward translation. Starting from the original English version developed by Bernstein et al. (2003), a bilingual psychologist, a native Spanish speaker with a command of English and knowledge of the Peruvian sociocultural context, translated the 28 items of the CTQ-SF into Peruvian Spanish. The original items published by Bernstein et al. (2003) are freely available in the literature, so no direct authorization was required.

Stage 2. Back-translation. An independent language teacher with an advanced command of English, blinded to the original version, performed the back-translation of the instrument from Spanish into English, in order to verify the correspondence between the translated version and the original.

Stage 3. Consensus and reconciliation. The two translators together with the research team held a virtual consensus meeting via Zoom, during which the original, translated, and back-translated versions were compared item by item. Discrepancies, as well as lexical and comprehensibility aspects, were discussed until agreement was reached that each item preserved the meaning of the original and was clear for the target population, thereby consolidating the preliminary Peruvian Spanish version of the CTQ-SF. This version was subsequently subjected to expert content validation (see Data analysis and Results), whereas no pilot testing was conducted. The full item-by-item correspondence between the original and adapted versions, and the final 18-item version with scoring instructions, are provided in Supplementary Tables S1, S2.

### 2.5 Procedure

The study was reviewed and approved by the Research Ethics Committee of the Graduate School of Universidad Peruana Unión. Once ethical authorization was obtained, the data collection phase proceeded following a previously established schedule. Before data collection began, an informed consent document was distributed to the parents or legal guardians of the students, clearly describing the study objectives, the procedures to be followed, and the measures implemented to protect data privacy and confidentiality. After obtaining parental consent, informed assent was requested from the participating students. Both documents were written in accessible language, ensuring that parents and students fully understood the scope and nature of their voluntary participation. The instruments were administered in person under standardized conditions for all participants. The researchers acted as facilitators, coordinating and supervising the distribution of the instruments and ensuring that administration conditions were uniform and appropriate.

### 2.6 Data analysis

Content validity was analyzed using Aiken’s *V* coefficient (Aiken, 1985), computing 95% confidence intervals (Penfield and Giacobbi, 2004). Items were accepted when the lower bound was ≥0.50, with no need for further modifications.

Regarding statistical processing, R software (version 4.1) (R Core Team, 2021) was used through the Jamovi interface (version 2.6.44) (The jamovi project, 2023). Within this approach, the R package lavaan (Rosseel, 2012) was used, implemented in the SEMLj module of Jamovi (Gallucci and Jentschke, 2021). The dataset was randomly split into two subsamples: 439 participants for the EFA and 439 for the CFA. The random split was performed using the simple random-number function in Microsoft Excel, ensuring that no participant belonged to both subsamples.

Prior to the EFA, statistical assumptions were checked. Univariate normality was assessed through skewness and kurtosis values. Sampling adequacy was verified with the Kaiser–Meyer–Olkin (KMO) index, where high values indicated the suitability of factor analysis (Kaiser, 1974). Bartlett’s test of sphericity, which tests the null hypothesis of independence among variables (i.e., an identity correlation matrix), was also applied. For the CFA, skewness and kurtosis indices were likewise examined. The marked skewness and kurtosis of the items justified the use of polychoric correlations and the WLSMV estimator, both appropriate for non-normal ordinal data.

The CTQ-SF also includes a three-item Minimization/Denial validity scale (items 10, 16, and 22) designed to detect the tendency to underreport maltreatment. These three items were administered but were excluded from the exploratory and confirmatory factor analyses, as they assess a response bias rather than a maltreatment construct. None of these validity items is part of the final 18-item version. Accordingly, the factorial structure reported here is based on the 25 clinical items, and the final validated version comprises 18 items distributed across three factors.

Missing data were handled through listwise deletion, as only complete cases were included in the analyses. The seven positively worded items reflecting perceived family support (items 2, 5, 7, 13, 19, 26, and 28) were reverse-scored prior to all factor analyses, so that higher scores consistently indicated greater exposure to maltreatment. The EFA was performed using the minimum residuals method with oblimin rotation, based on polychoric correlations, given the ordinal nature of the items, applying a retention criterion of factor loadings above 0.40 (Lloret-Segura et al., 2014). Items were retained if their primary loading was ≥0.40 and did not present cross-loadings ≥0.30 on more than one factor. The number of factors was determined through parallel analysis, widely recommended in the literature (Lloret-Segura et al., 2014), which selects factors whose eigenvalues exceed those obtained at random (Horn, 1965) using 20 random datasets and the 95th percentile of the randomly generated eigenvalues as the retention threshold. Subsequently, the CFA was conducted with the lavaan package, applying the WLSMV estimator, which is appropriate for ordinal data (Likert-type items) and robust against violations of multivariate normality (Finney and DiStefano, 2013). Model goodness of fit was evaluated with the chi-square test (*χ*^2^), the comparative fit index (CFI), the Tucker–Lewis index (TLI), the root mean square error of approximation (RMSEA), and the standardized root mean square residual (SRMR), accepting as criteria CFI and TLI > 0.90 (Hooper et al., 2008); for RMSEA and SRMR, values <0.05 were considered good fit and <0.08 acceptable (Bandalos and Finney, 2019; Kline, 2016).

Regarding reliability, ordinal versions of Cronbach’s alpha and McDonald’s omega (total omega) were computed from the polychoric correlation matrix, given the ordinal nature of the items, using the psych package (Revelle, 2024) in R, accepting values above 0.70 as adequate (Cheung et al., 2024). Factor loadings (*λ*) were also appraised, with values above 0.70 considered satisfactory. Regarding construct validity, convergent validity was estimated through the average variance extracted (AVE), where values above 0.50 were interpreted as adequate (Fornell and Larcker, 1981).

### 2.7 Ethical considerations

Ethical approval for this study was obtained from the Research Ethics Committee of the Graduate School of Universidad Peruana Unión (approval No. 2025-CEEPG-00215). Informed consent was obtained from parents or legal guardians, and assent was obtained from all adolescent participants prior to data collection. To safeguard participants’ wellbeing, several protective measures were implemented. Before administration, school psychologists at each institution were briefed on the study and agreed to provide psychological evaluation and follow-up to any student who showed signs of emotional distress as a result of participation. During administration, participants were informed that they could withdraw from the questionnaire at any time if they felt uncomfortable or distressed, without any negative consequences; any student who reported feeling affected by the content was allowed to discontinue immediately and was referred to the school psychologist for timely psychological evaluation and support. Confidentiality of all responses was strictly maintained throughout the process. In accordance with Peruvian child protection legislation (Decreto Legislativo N° 1297), school psychologists were informed of their mandatory reporting obligations in the event that any participant disclosed an ongoing abuse situation requiring intervention by child protection authorities.

The informed consent obtained from parents and legal guardians covered the use of anonymized data for research purposes; the ethics committee approved data sharing on the condition that individual participants could not be identified.

## 3 Results

### 3.1 Sociodemographic characteristics

Table 1 presents the sociodemographic data of the participants, showing a predominance of female sex (54.8%), ages between 12 and 17 years (M = 14.0; SD = 1.46), living with both parents and siblings (71.4%), and in the first year of secondary education (33.8%).

**Table 1:** Sociodemographic characteristics of the participants.

Characteristic	Category	Frequency	%
Sex	Female	481	54.8
	Male	397	45.2
With whom do you live?	Both parents and siblings	627	71.4
	Both parents only	155	17.7
	Others	96	10.9
Grade	First year	297	33.8
	Second year	171	19.5
	Third year	176	20.0
	Fourth year	144	16.4
	Fifth year	90	10.3

### 3.2 Content validity

The content validity of the questionnaire was assessed by five expert judges (three clinical psychologists and two educational psychologists). The items were evaluated in terms of relevance, representativeness, and clarity. Each criterion was rated on a scale from 0 to 3. Minor wording adjustments were made to some items, detailed in the Supplementary material. Aiken’s *V* coefficient was computed, and all items obtained adequate values with lower confidence bounds ≥0.50 (Table 2).

**Table 2:** Content validity.

Items	Relevance		Representativeness		Clarity	
	*V*	95% IC	*V*	95% IC	*V*	95% IC
T1	0.93	(0.76–0.98)	0.93	(0.76–0.98)	0.93	(0.76–0.98)
T2	0.93	(0.76–0.98)	0.87	(0.68–0.95)	0.87	(0.68–0.95)
T3	0.93	(0.76–0.98)	0.87	(0.68–0.95)	0.93	(0.76–0.98)
T4	0.93	(0.76–0.98)	0.87	(0.68–0.95)	0.80	(0.60–0.91)
T5	0.87	(0.68–0.95)	0.80	(0.60–0.91)	0.87	(0.68–0.95)
T6	0.93	(0.76–0.98)	0.93	(0.76–0.98)	0.93	(0.76–0.98)
T7	0.87	(0.68–0.95)	0.93	(0.76–0.98)	0.93	(0.76–0.98)
T8	0.93	(0.76–0.98)	0.93	(0.76–0.98)	0.93	(0.76–0.98)
T9	0.93	(0.76–0.98)	0.93	(0.76–0.98)	1.00	(0.86–1.00)
T11	0.93	(0.76–0.98)	0.93	(0.76–0.98)	0.87	(0.68–0.95)
T12	0.93	(0.76–0.98)	1.00	(0.86–1.00)	0.93	(0.76–0.98)
T13	0.87	(0.68–0.95)	0.93	(0.76–0.98)	0.87	(0.68–0.95)
T14	0.80	(0.60–0.91)	0.87	(0.68–0.95)	0.80	(0.60–0.91)
T15	0.73	(0.53–0.87)	0.87	(0.68–0.95)	0.87	(0.68–0.95)
T17	0.80	(0.60–0.91)	0.80	(0.60–0.91)	0.93	(0.76–0.98)
T18	0.87	(0.68–0.95)	0.93	(0.76–0.98)	0.87	(0.68–0.95)
T19	0.87	(0.68–0.95)	0.93	(0.76–0.98)	0.93	(0.76–0.98)
T20	0.87	(0.68–0.95)	0.93	(0.76–0.98)	0.93	(0.76–0.98)
T21	0.80	(0.60–0.91)	0.87	(0.68–0.95)	0.93	(0.76–0.98)
T23	0.80	(0.60–0.91)	0.87	(0.68–0.95)	0.93	(0.76–0.98)
T24	0.87	(0.68–0.95)	0.73	(0.53–0.87)	0.73	(0.53–0.87)
T25	0.80	(0.60–0.91)	0.87	(0.68–0.95)	0.87	(0.68–0.95)
T26	0.73	(0.53–0.87)	0.93	(0.76–0.98)	0.93	(0.76–0.98)
T27	0.73	(0.53–0.87)	0.93	(0.76–0.98)	0.87	(0.68–0.95)
T28	0.80	(0.60–0.91)	0.93	(0.76–0.98)	0.93	(0.76–0.98)

### 3.3 Preliminary analysis

The descriptive analysis of the 28 items was carried out independently in the two subsamples: the EFA subsample (*n* = 439) and the CFA subsample (*n* = 439). Several items showed high skewness and kurtosis values, particularly those referring to severe forms of maltreatment such as severe physical abuse (T4) and sexual abuse (T9), with distributions markedly skewed toward the low end of the scale. This pattern is expected and consistent with the nature of the phenomenon under study: severe maltreatment experiences are low-frequency events in the general population, so most participants tend to endorse the lowest category, producing strong floor effects (Bernstein et al., 2003; Kline, 2016). Because the assumption of multivariate normality was not met, and given the ordinal nature of the items, the confirmatory and invariance analyses were conducted using the weighted least squares mean- and variance-adjusted estimator (WLSMV), which does not assume normality and is more appropriate than maximum likelihood for ordinal categorical data with skewed distributions (Li, 2016; Wu and Estabrook, 2016). The complete descriptive statistics are presented in Table 3.

**Table 3:** Preliminary item analysis.

Item	EFA *n* = 439				CFA *n* = 439			
	Mean	SD	*g*1	*g*2	Mean	SD	*g*1	*g*2
T1	1.39	0.77	2.08	3.76	1.37	0.76	2.35	5.82
T2	2.14	1.26	0.80	−0.52	2.16	1.27	0.79	−0.57
T3	2.05	1.10	0.87	−0.01	1.95	1.03	0.94	0.32
T4	1.13	0.58	5.42	30.57	1.09	0.50	6.57	46.15
T5	2.39	1.29	0.65	−0.67	2.42	1.26	0.58	−0.63
T6	1.32	0.87	3.03	8.65	1.3	0.86	3.29	10.40
T7	2.22	1.32	0.71	−0.74	2.19	1.27	0.82	−0.42
T8	1.53	1.04	2.11	3.59	1.49	1.05	2.22	3.92
T9	1.2	0.63	3.57	13.15	1.23	0.77	3.73	13.59
T10	2.15	1.40	0.86	−0.66	2.09	1.40	1.01	−0.39
T11	1.5	0.96	2.16	4.18	1.51	0.93	2.05	3.87
T12	2	1.16	1.00	0.07	1.92	1.06	1.09	0.67
T13	2.37	1.40	0.68	−0.85	2.32	1.35	0.75	−0.65
T14	1.71	1.12	1.59	1.59	1.62	1.05	1.77	2.31
T15	1.23	0.74	3.58	12.58	1.15	0.61	4.61	22.67
T16	3.27	1.47	−0.31	−1.31	3.23	1.44	−0.20	−1.33
T17	1.23	0.66	3.44	12.71	1.18	0.65	4.15	18.10
T18	1.56	0.97	1.86	2.92	1.45	0.93	2.22	4.31
T19	2.37	1.35	0.72	−0.68	2.39	1.38	0.66	−0.86
T20	1.28	0.83	3.29	10.24	1.26	0.80	3.62	12.93
T21	1.21	0.73	3.81	14.28	1.15	0.65	4.83	23.76
T22	3.77	1.41	−0.81	−0.74	3.77	1.38	−0.80	−0.68
T23	1.15	0.63	4.96	25.27	1.13	0.53	5.26	30.81
T24	1.23	0.76	3.70	13.57	1.2	0.66	3.96	16.92
T25	1.3	0.76	2.93	8.83	1.3	0.74	3.09	10.42
T26	2.3	1.35	0.72	−0.71	2.32	1.39	0.76	−0.73
T27	1.09	0.51	6.16	39.21	1.11	0.54	5.72	34.30
T28	1.9	1.19	1.15	0.17	1.94	1.25	1.19	0.25

EFA, exploratory factor analysis sample; CFA, confirmatory factor analysis sample; M, mean; SD, standard deviation; *g*1, skewness; *g*2, kurtosis.

### 3.4 Comparison of 5 vs. 3 factors

Given that the parallel analysis in the EFA did not support the extraction of five factors—the fourth factor contained no items with sufficient own loadings (only T18 loaded weakly and cross-loaded), and no fifth factor emerged—the original five-factor structure could not be reproduced exploratorily. To empirically document this, the original five-factor model (25 clinical items) was estimated confirmatorily using its theoretical item assignment and compared descriptively with the three-factor solution (18 items). The five-factor model showed poorer fit (CFI = 0.919; TLI = 0.908; SRMR = 0.086; RMSEA = 0.054) than the three-factor model (CFI = 0.958; TLI = 0.951; SRMR = 0.067; RMSEA = 0.051), and its physical-neglect factor exhibited inadequate reliability and convergent validity (F4: *α* = 0.423; *ω* = 0.495; AVE = 0.260) (Table 4). Because the two models involve different sets of observed variables, they are not statistically nested; the comparison is therefore descriptive.

**Table 4:** Comparison of 5 vs. 3 factors.

Fit indices	5-factor model	3-factor model
*χ*^2^ (df)	604 (265)	282 (132)
CFI	0.919	0.958
TLI	0.908	0.951
SRMR	0.086	0.067
RMSEA	0.054	0.051
95% CI RMSEA	0.048–0.060	0.043–0.059
RMSEA (p)	0.117	0 0.415
AVE	F1 = 0.521	F1 = 0.418
	F2 = 0.512	F2 = 0.808
	F3 = 0.809	F3 = 0.502
	F4 = 0.260	
	F5 = 0.451	
*α* ordinal	F1 = 0.657	F1 = 0.824
	F2 = 0.710	F2 = 0.952
	F3 = 0.860	F3 = 0.841
	F4 = 0.423	
	F5 = 0.729	
*ω*	F1 = 0.696	F1 = 0.795
	F2 = 0.742	F2 = 0.880
	F3 = 0.878	F3 = 0.757
	F4 = 0.495	
	F5 = 0.759	
HTMT	Entre: 0.263–0.867	Entre: 0.262–0.626

### 3.5 Internal structure

The subsample (*n* = 439) was deemed adequate for the exploratory factor analysis (EFA) according to the Kaiser–Meyer–Olkin (KMO) measure of sampling adequacy and Bartlett’s test of sphericity. The KMO value was 0.836, indicating adequate sampling adequacy, and Bartlett’s test of sphericity was significant (*χ*^2^ = 2,437, df = 153, *p* < 0.001). The number of factors was determined through parallel analysis and the scree plot (Figure 2). Although the initial parallel analysis suggested four factors, this fourth factor lacked its own items with sufficient loadings; therefore—on the basis of interpretability, parsimony, and theoretical coherence—a three-factor solution was retained. The EFA was conducted using the minimum residuals extraction method and Oblimin rotation, retaining items with a factor loading ≥0.40. After removing items with insufficient loadings (T1, T3, T4, T6, T18), items with content redundancy (T15 and T25), and the validity items (T10, T16, T22), which showed negative loadings on other factors, an 18-item structure organized into three factors was confirmed (Table 5), labeled: lack of perceived family support/neglect (Factor 1), sexual abuse (Factor 2), and physical–emotional abuse (Factor 3). Items T15 and T25 are general, summary-type indicators of perceived abuse (‘I was physically abused’ and ‘I was emotionally abused’) that adolescents—many still in cognitive and emotional development—tend to interpret as almost identical, which introduces content redundancy between them; by contrast, item T27 (‘I was sexually abused’) did not present this problem, as its content is clearly distinct in nature and severity. The three-factor solution accounted for 40.8% of the total variance. Although the overall solution showed adequate fit, three retained items presented relatively low communalities: T2 (*h*^2^ = 0.26), T9 (*h*^2^ = 0.24), and T21 (*h*^2^ = 0.28), indicating that a notable portion of their variance was not accounted for by the three-factor structure.

**Figure 2:**
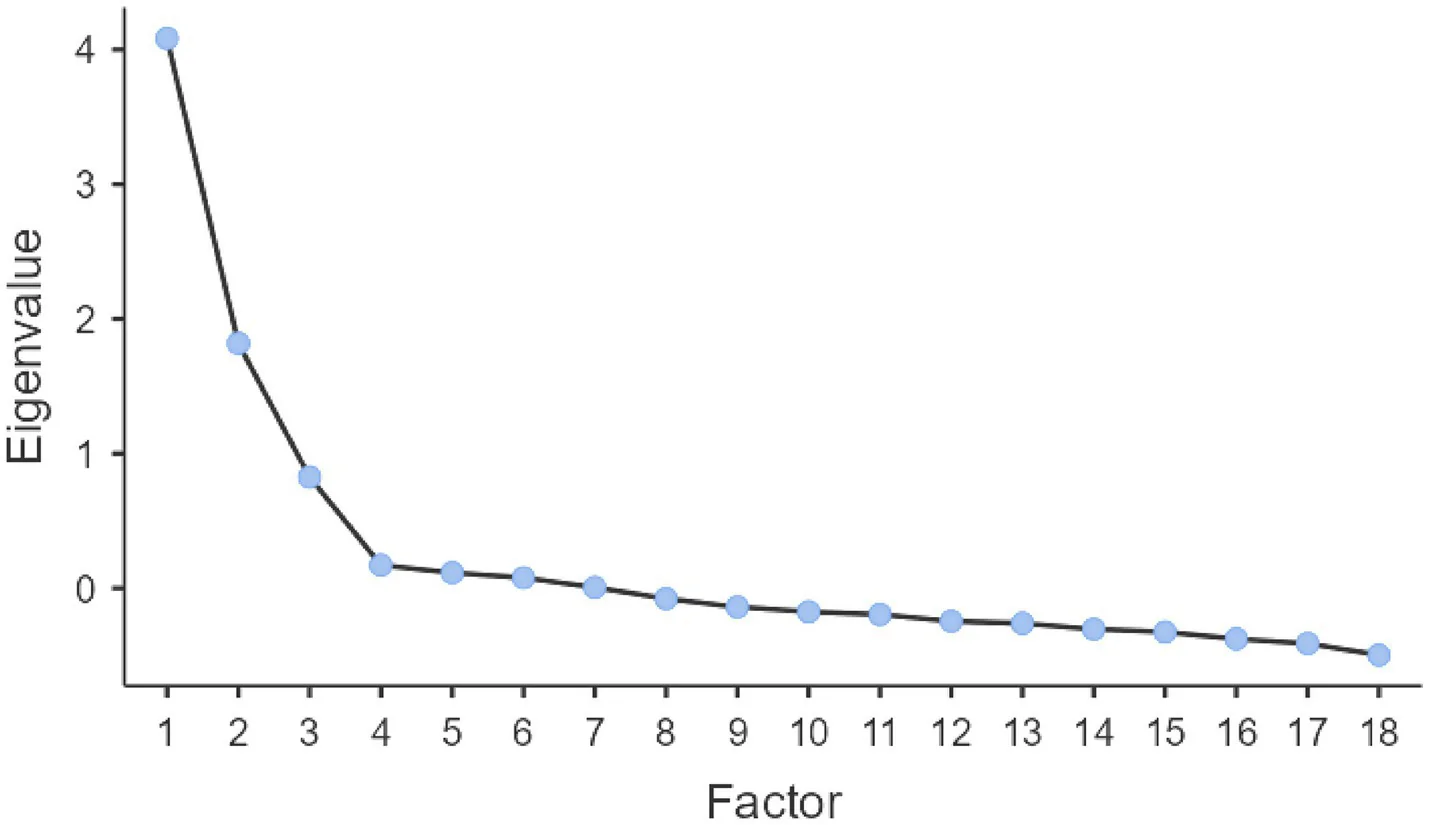
Scree plot of the CTQ-SF.

**Table 5:** Exploratory and confirmatory factor analysis of the items.

Items	F1	F2	F3	*h* ^2^	F1	F2	F3	Items
T2	0.51			0.26	0.53			T2
T5	0.64			0.36	0.47			T5
T7	0.70			0.53	0.56			T7
T13	0.56			0.32	0.71			T13
T19	0.68			0.48	0.81			T19
T26	0.55			0.30	0.64			T26
T28	0.58			0.40	0.73			T28
T20		0.69		0.50		0.92		T20
T21		0.48		0.28		0.87		T21
T23		0.77		0.58		0.89		T23
T24		0.77		0.59		0.91		T24
T27		0.78		0.60		0.90		T27
T8			0.42	0.28			0.68	T8
T9			0.51	0.24			0.63	T9
T11			0.76	0.54			0.70	T11
T12			0.56	0.33			0.66	T12
T14			0.49	0.44			0.77	T14
T17			0.46	0.32			0.80	T17
*α*	0.800	0.818	0.736		0.775	0.860	0.721	*α*
*ω*	0.803	0,834	0.747		0.824	0.952	0.841	*α* ordinal
					0.795	0.880	0.757	*ω*
					0.418	0.808	0.502	AVE
					0.864	0.956	0.866	*H*
					1.000	0.430	0.390	F1
					0.430	1.000	0.810	F2
					0.390	0.810	1.000	F3
					*F1*	*F2*	*F3*	
				*F1*	1.000	0.314	0.262	*HTMT*
				*F2*	0.314	1.000	0.626	
				*F3*	0.262	0.626	1.000	

EFA, exploratory factor analysis sample; CFA, confirmatory factor analysis sample; *h*^2^, communality; AVE, average variance extracted; HTMT, heterotrait–monotrait ratio. F1 = lack of perceived family support/neglect; F2 = sexual abuse; F3 = physical–emotional abuse.

### 3.6 Internal structure validation

A confirmatory factor analysis (CFA) was conducted on the independent subsample (*n* = 439) to evaluate the three correlated-factor model of the CTQ-SF, comprising 18 items. Given the ordinal nature of the items, the WLSMV estimator was used. The model showed an adequate fit to the data: *χ*^2^(132) = 282, *p* < 0.001; CFI = 0.958; TLI = 0.951; RMSEA = 0.051 (95% CI 0.043–0.059); SRMR = 0.067. The standardized factor loadings ranged from 0.47 to 0.92 and were statistically significant in all cases (*p* < 0.001) (Figure 3). No *post hoc* modifications (e.g., correlated residuals) were introduced; the model tested corresponded exactly to the three-factor structure derived from the EFA.

**Figure 3:**
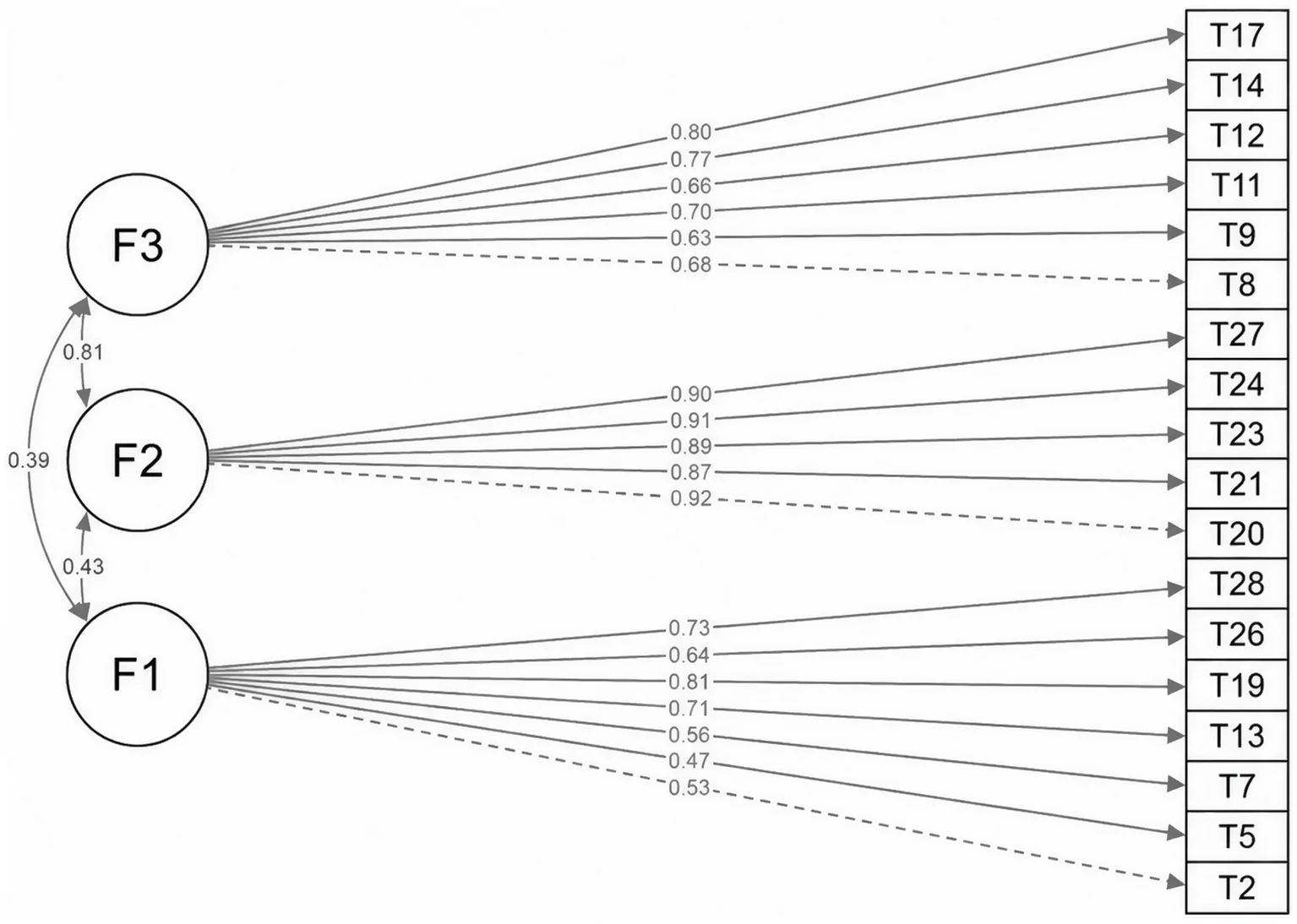
Structural diagram of the three-factor model of the CTQ-SF.

The internal consistency of each dimension was adequate, with ordinal alpha coefficients of 0.824 (lack of perceived family support/neglect), 0.952 (Sexual abuse), and 0.841 (Physical–emotional abuse), and McDonald’s omega coefficients of 0.795, 0.880, and 0.757, respectively, exceeding the 0.70 criterion (Cheung et al., 2024). The average variance extracted (AVE) values were 0.418 (lack of perceived family support/neglect), 0.808 (Sexual abuse), and 0.502 (Physical–emotional abuse). Although the AVE of the neglect dimension fell below the 0.50 threshold, its coefficient *H* (Table 5) exceeded 0.60, so convergent validity is considered adequate. Discriminant validity was assessed through the heterotrait–monotrait ratio (HTMT); all values were below 0.85 (range 0.262–0.626), supporting the empirical distinction among the three factors (Henseler et al., 2015) (Table 5). The Fornell–Larcker criterion was also met, as the square root of each factor’s AVE (0.65, 0.90, and 0.71) exceeded its correlations with the other factors (Fornell and Larcker, 1981).

### 3.7 Factorial invariance

Measurement invariance was assessed between males (*n* = 397) and females (*n* = 481) through multigroup CFA with the WLSMV estimator (Wu and Estabrook, 2016), after collapsing the two highest response categories (4 = Often and 5 = Very often) of item T27 (‘I was sexually abused’), the only item affected, because of their low endorsement; the same recoding was applied to both sex groups. In the scalar model, factor loadings and item thresholds were constrained to equality across groups, as recommended for ordinal indicators with the WLSMV estimator. The configural model showed good fit (CFI = 0.962; RMSEA = 0.049; SRMR = 0.086), and the successive constraints (metric and scalar) did not deteriorate the fit. Although the *χ*^2^ difference tests were significant, the incremental fit indices were prioritized owing to their sensitivity to sample size (Cheung and Rensvold, 2002): all differences remained below the thresholds (ΔCFI ≤ 0.010; ΔRMSEA ≤ 0.015; Chen, 2007). Full scalar invariance was thus established, which enables the comparison of latent means across sexes (Table 6).

**Table 6:** Measurement invariance across sex (scaled values).

Model	*χ*^2^ (df)	CFI	RMSEA (IC 90%)	SRMR	Model Comp.	*Δχ*^2^ (*Δ*df)	*Δ*CFI	*Δ*RMSEA	Decision
M1: configural	538.76 (264)	0.962	0.049 (0.043–0.059)	0.086	—	—	—	—	Accept
M2: metric	588.30 (305)	0.961	0.046 (0.041–0.052)	0.087	M1	60.07 (41)^*^	−0.001	−0.003	Accept
M3: scalar	620.19 (320)	0.959	0.046 (0.041–0.051)	0.090	M2	50.13 (15)^***^	−0.002	0.000	Accept

*p < 0.05; ***p < 0.001.

### 3.8 Comparison of latent means by sex

Because scalar invariance was established, latent means were compared across sexes using the male group as the reference. Females reported significantly higher levels of sexual abuse (*Δ* = 1.21, *z* = 4.94, *p* < 0.001), with a large effect size. No significant differences were found in neglect (*Δ* = 0.11, *z* = 1.32, *p* = 0.187) or in physical–emotional abuse (*Δ* = 0.22, *z* = 1.87, *p* = 0.062), although in the latter dimension a trend toward higher scores in females was observed. These results are consistent with epidemiological evidence documenting a higher prevalence of childhood sexual abuse in females.

## 4 Discussion

This study aimed to adapt and analyze the psychometric properties of the Childhood Trauma Questionnaire–Short Form (CTQ-SF) in adolescents from public schools in the San Martín Region, Peru. The results showed that the original five-factor structure was not replicated; instead, a three-factor correlated solution emerged—lack of perceived family support/neglect, sexual abuse, and physical–emotional abuse—which showed adequate validity evidence based on its internal structure, reliability, and measurement invariance across sex.

### 4.1 The five-factor structure and its failure to replicate

The original five-factor model of Bernstein et al. (2003) was not supported in this study. Far from representing an anomaly, this finding is consistent with the literature indicating that the original CTQ-SF structure is difficult to reproduce stably across languages and cultural contexts (Georgieva et al., 2021). Indeed, in the early factorial studies of the instrument, the physical and emotional abuse items already loaded onto a single factor (Bernstein et al., 1994), and subsequent systematic reviews have documented that four- and three-factor solutions, as well as bifactor models, provide fit comparable or superior to the five-factor model across diverse populations (Georgieva et al., 2021). This difficulty in replication is reinforced by the findings of the most comprehensive reliability generalization meta-analysis of the CTQ-SF to date, which, drawing on 39 independent samples and 243 reliability coefficients, found broad and statistically significant heterogeneity (*I*^2^ between 96.4 and 98.6%) in the alpha coefficients of the five original subscales—heterogeneity that could not be explained by sampling error alone and that suggests the five-factor structure does not behave uniformly across populations and cultural contexts (Badenes-Ribera et al., 2024). Because the final version comprised 18 items rather than the 25 clinical items of the original model, no direct statistical comparison was made between the two structures, as they do not operate on the same set of observed variables; instead, the three-dimensional structure was supported by the empirical evidence derived from the EFA and confirmed through CFA in an independent subsample. Four-factor and bifactor models were not evaluated either, given that the parallel analysis provided no empirical support for a fourth factor.

### 4.2 Convergence of physical and emotional abuse

The empirical collapse of physical and emotional abuse into a single factor is not a sample artifact but a widely documented pattern. Studies in non–North American samples have recurrently reported models that combine both domains into a single dimension of active abuse (Cuomo et al., 2008; Grassi-Oliveira et al., 2014). This convergence is explained by the fact that both forms of maltreatment tend to co-occur within the same family environment and share common psychological and contextual mechanisms, which hinders their empirical differentiation when assessed through retrospective self-report (Higgins and McCabe, 2001; Stoltenborgh et al., 2013). Theoretically, this grouping is consistent with the classical distinction between acts of commission—encompassing physical, emotional, and sexual abuse—and acts of omission—neglect—established by the World Health Organization (Laajasalo et al., 2023), as well as with dimensional approaches to maltreatment that organize its manifestations into the emotional, physical, and sexual domains (Baldwin et al., 2023). This convergence also finds indirect support in the reliability generalization meta-analysis of Badenes-Ribera et al. (2024): the physical abuse [*α* = 0.843, 95% CI (0.818, 0.865), *k* = 36] and emotional abuse [*α* = 0.839, 95% CI (0.819, 0.857), *k* = 36] subscales showed virtually identical reliability coefficients across 36 independent samples, and both dimensions shared the same statistically significant moderators—score variability, the geographical location of the study, and the funding source—suggesting that both domains follow very similar psychometric patterns and may not be as empirically distinguishable as the original five-factor model proposes.

### 4.3 The specificity of sexual abuse

Unlike the other dimensions, sexual abuse retained its specificity, showing the highest factor loadings and the greatest internal consistency of the instrument. This finding converges with that of a recent bifactor model of the CTQ-SF, in which only sexual abuse separated from a general factor of intrafamilial maltreatment across two independent French-speaking samples (Delhalle et al., 2024). The specificity of sexual abuse is theoretically expected: it constitutes a qualitatively distinct form of victimization, with lower co-occurrence with other types of maltreatment and a particular phenomenological nature (Dong et al., 2003), which explains its stable empirical differentiation across studies. This specificity is reinforced by the evidence accumulated in the meta-analysis of Badenes-Ribera et al. (2024): of the five CTQ-SF subscales, sexual abuse was consistently the one showing the highest and most stable average reliability, both in internal consistency [alpha = 0.916, 95% CI (0.896, 0.931), *k* = 37; omega = 0.900, *k* = 2] and in temporal stability (*r* = 0.677, *k* = 4), clearly surpassing the other dimensions across 37 independent samples drawn from highly diverse clinical, community, and cultural contexts. This cross-cultural robustness of sexual abuse, together with the evidence from the bifactor model noted above (Delhalle et al., 2024), converges with the present finding that this dimension remained unaltered within the three-dimensional structure obtained.

### 4.4 The merging of the neglect dimensions into lack of perceived family support/neglect

The physical and emotional neglect dimensions merged into a single factor, another recurrent pattern. Several studies have proposed collapsing both subscales into a single neglect dimension, arguing that the concepts of physical and emotional neglect in the original model are too intertwined to be distinguished empirically (Peng et al., 2023). In particular, the physical neglect subscale has repeatedly been identified as the one with the lowest internal consistency and the weakest factor loadings across different cultural contexts (García-Fernández et al., 2024; Kongerslev et al., 2019), a pattern also observed in the present sample. The integration of both types of neglect into a single, more consistent factor therefore represents an empirically more robust solution than the original separation.

This pattern fully coincides with the large-scale reliability generalization study of Badenes-Ribera et al. (2024), which constitutes the most robust evidence available on the psychometric behavior of the CTQ-SF internationally: of the five original subscales, physical neglect was consistently the least reliable, both in internal consistency [alpha = 0.656, 95% CI (0.606, 0.698), *k* = 36, with individual estimates ranging from 0.36 to 0.87; omega = 0.740, *k* = 3] and in temporal stability (*r* = 0.668, *k* = 5), whereas emotional neglect showed considerably higher reliability [alpha = 0.851, 95% CI (0.827, 0.872), *k* = 35; omega = 0.914, *k* = 4]. According to the criteria of Cicchetti (1994), an alpha of 0.656 falls within the “unacceptable” range for clinical or exploratory research, whereas the mean alpha of emotional neglect falls within the “good” range. This asymmetry between the two neglect subscales has also been documented in adolescent samples from other contexts: in a representative sample of more than 20,000 Chinese adolescents, Peng et al. (2023) found that an alternative four-factor model, in which the physical and emotional neglect items were collapsed into a single neglect subscale, showed acceptable fit and notably higher internal consistency (*α* = 0.824 for the combined subscale) than physical neglect assessed in isolation (*α* = 0.491)—a result that strikingly coincides with the pattern found in the present Peruvian sample. Likewise, in the Spanish validation by García-Fernández et al. (2024) with adolescents at risk of suicide, physical neglect was again the only CTQ-SF subscale that did not reach acceptable levels of internal consistency (alpha = 0.624), even though the remaining dimensions—including emotional neglect—did. Earlier, Georgieva et al. (2021), in their systematic review of ten CTQ-SF validations, had noted that physical neglect was the subscale that most consistently showed reliability below 0.70, with occasional additional problems in emotional neglect, emotional abuse, and physical abuse.

Badenes-Ribera et al. (2024) further offer a substantive—not merely statistical—explanation for this pattern, which is particularly relevant for interpreting the present findings in a Latin American context. The authors note that the concept of physical neglect may be culturally sensitive, since some of its items (e.g., “there was not enough to eat,” “there was someone to take me to the doctor if I needed it,” or “I wore dirty clothes”) may not reflect intentional maltreatment but rather the economic and cultural conditions of the environment, which would reduce the covariances with the remaining subscale indicators and produce a loss of unidimensionality—and, consequently, lower reliability—particularly in countries outside the North American context in which the instrument was originally developed. Consistent with this explanation, the meta-analysis’s moderator analysis showed that the reliability of the total CTQ-SF and several of its subscales was systematically lower in studies conducted in Asia and Africa than in Europe and North America (Badenes-Ribera et al., 2024), underscoring the importance of examining the behavior of physical neglect with particular care in non-Western populations, such as the Peruvian adolescents assessed in this study. The empirical integration of both types of neglect into a single, more consistent factor observed in the present research can thus be understood not only as a statistically more robust solution but also as a reasonable response to a structural limitation of the physical neglect item widely documented in the international literature. It should be noted that this factor is composed of positively worded, reverse-scored items, and therefore represents the absence of family support (i.e., emotional neglect) rather than a direct experience of maltreatment; a method effect associated with the positive wording cannot be ruled out, an aspect that future studies could examine through a model incorporating a method factor.

### 4.5 Reliability, convergent validity, and discriminant validity

The three dimensions attained adequate reliability coefficients (ordinal alpha and omega above 0.75). The AVE of the neglect dimension fell slightly below the 0.50 threshold; nevertheless, since its coefficient *H* exceeded 0.60, convergent validity can be considered adequate (Fornell and Larcker, 1981). This nuance is expected in a factor that integrates two subdomains of heterogeneous content. Notably, the reliability obtained in the present study for the neglect dimension was markedly higher than the international average reported for physical neglect assessed in isolation (omega = 0.740; Badenes-Ribera et al., 2024), reinforcing the idea that merging both types of neglect produces a psychometrically more solid indicator than its separate components. Discriminant validity was supported by HTMT values below 0.85 in all cases, confirming that the three dimensions, although correlated, are empirically distinguishable (Henseler et al., 2015). It is worth distinguishing these three levels of evidence: adequate global factorial fit does not, by itself, guarantee high internal consistency in each dimension or full construct validity, and each was therefore assessed independently.

It should be noted that three retained items (T2, T9, and T21) showed relatively low communalities (*h*^2^ < 0.30), suggesting that the three-factor structure does not fully capture the variance of these indicators. This may reflect the heterogeneity of the constructs they represent—perceived care/protection, physical severity, and sexual threat—or item-specific variance not shared with their respective factors. Future studies should examine whether these items maintain adequate psychometric performance across different samples and consider whether their retention is justified by their theoretical relevance to the construct.

### 4.6 Measurement invariance across sex

A relevant contribution of this study, absent from most previous CTQ-SF adaptations, was the establishment of full scalar invariance across sex. This result indicates that the instrument measures the construct in the same way in males and females and enables the valid comparison of latent scores between the two groups (Putnick and Bornstein, 2016). This scarcity of invariance studies is consistent with the limitations noted by Georgieva et al. (2021) and with the fact that the meta-analysis by Badenes-Ribera et al. (2024) itself could not formally examine this property owing to the small number of studies reporting it, which underscores the added value of the present research. In line with this, females reported significantly higher levels of sexual abuse, a finding consistent with epidemiological evidence documenting a higher prevalence of childhood sexual abuse in the female population (Finkelhor et al., 2014). The demonstration of invariance strengthens the usefulness of the instrument both for research and for applied practice in contexts requiring comparisons across sexes.

### 4.7 Practical implications

In applied terms, the three-factor structure identified offers a parsimonious and psychometrically sound configuration that can be used as a screening tool in educational and health settings, facilitating the early detection of adolescents exposed to maltreatment. Its brevity and the robustness of its indicators make it especially useful in resource-limited settings, where brief and valid instruments are needed to guide preliminary screening. Nevertheless, because criterion validity and diagnostic capacity were not evaluated, its use should for now be restricted to research and preliminary screening purposes and not to individual clinical decision-making.

### 4.8 Limitations

Although the study was conducted under rigorous methodological procedures, it has some limitations. First, the cross-sectional design precludes causal conclusions and the evaluation of temporal stability, so future studies should consider incorporating longitudinal designs. Second, as a retrospective, self-report instrument, the results may be subject to the biases characteristic of this type of measurement, such as memory bias, social desirability, and the minimization or denial of maltreatment experiences. Third, the sample comprised school-attending adolescents from the Peruvian Amazon (San Martín Region), which limits the generalizability of the results to non-schooled adolescents, private-school students, clinical populations, or other regions of the country. Fourth, reducing the instrument to a final 18-item version entails the exclusion of a considerable number of original items; although this decision was empirically justified, it precludes a direct statistical comparison with the classical five-factor structure. Fifth, measurement invariance was examined only across sex; future studies should evaluate invariance across age and other variables, as well as differential item functioning (DIF), which was not analyzed here. Sixth, the lack of perceived family support/neglect dimension, although it attained acceptable coefficient *H*, showed an AVE value slightly below the recommended threshold, consistent with its nature as a heterogeneous factor integrating physical and emotional neglect content. Seventh, data were collected in two schools, with students nested within classrooms; although this clustered structure could introduce some dependence among observations, the small number of schools (two) and the absence of a classroom identifier precluded estimating the intraclass correlation or applying multilevel models. This potential effect should be considered when interpreting the results, so future studies with more schools could apply multilevel approaches or cluster-robust standard errors. Eighth, because several items of the lack of perceived family support/neglect factor are positively worded, a wording-related method effect cannot be ruled out. Finally, the evidence provided pertains to the internal structure and internal consistency of the instrument; external criterion validity, and convergent and discriminant validity based on relationships with independent external measures, predictive validity, and temporal stability were not evaluated, and these remain pending for future research.

## 5 Conclusion

The present study provides the first psychometric evidence on the CTQ-SF in adolescents from public schools in the San Martín Region, Peru: a three-factor correlated model was established—lack of perceived family support/neglect, sexual abuse, and physical–emotional abuse—which, independently confirmed through EFA and CFA, showed adequate reliability, convergent and discriminant validity, and full scalar invariance across sex, evidence that is consistent with the most robust international psychometric literature on the instrument. These results provide preliminary evidence that this three-factor configuration constitutes a brief and promising alternative, with adequate internal consistency, for the study of childhood maltreatment in adolescents from the Peruvian Amazon. Nevertheless, it requires independent replication and the evaluation of its temporal stability and external validity before use in applied settings, so for now its use should be restricted to research and preliminary screening purposes. The scalar invariance across sex constitutes a strength that supports its usefulness for between-group comparisons.

## Data Availability

The datasets presented in this study can be found in online repositories. The names of the repository/repositories and accession number(s) can be found in the article/Supplementary material.

## Appendix

Final version of the Childhood Trauma Questionnaire–Short Form (CTQ-SF) adapted to the sample of adolescents from the Peruvian Amazon (items are presented in the Spanish version administered)

**Table:**

Nunca es cierto	Rara vez es cierto	A veces es cierto	Frecuentemente es cierto	Muy frecuentemente es cierto
1	2	3	4	5

**Table:**

No.	Item	Coding
Factor 1: Falta de apoyo familiar percibido/abandono (7 ítems)		
T2	Tuve a alguien que me cuidaba y protegía	Reverse (R)
T5	Alguien me ayudó a sentirme importante	Reverse (R)
T7	Me sentía amado/a	Reverse (R)
T13	Mi familia se cuidaba entre sí	Reverse (R)
T19	Mi familia se sentía unida entre sí	Reverse (R)
T26	Alguien me llevó al médico cuando lo necesitaba	Reverse (R)
T28	Mi familia me dio fuerza y apoyo	Reverse (R)
Factor 2: Abuso sexual (5 ítems)		
T20	Alguien intentó tocarme sexualmente o hacer que yo lo/la tocara	Direct
T21	Alguien me amenazó con hacerme daño si no hacía algo sexual con él/ella	Direct
T23	Alguien me obligó a hacer cosas sexuales o a ver cosas sexuales	Direct
T24	Alguien me tocó de manera inusual o inapropiada	Direct
T27	Fui abusado/a sexualmente	Direct
Factor 3: Abuso físico y emocional (6 ítems)		
T8	Sentía o pensaba que mis padres deseaban que yo no hubiera nacido	Direct
T9	Me golpearon tan fuerte que tuve que ver a un médico	Direct
T11	Me golpearon tan fuerte que me dejaron moretones o marcas	Direct
T12	Me castigaron con un cinturón, tabla, cable u otro objeto duro	Direct
T14	Mi familia me decía cosas hirientes o insultantes	Direct
T17	Me golpearon tan fuerte que lo notó un maestro, vecino y/o médico	Direct
