# Supplementary material for: Psychometric properties of the Childhood Trauma Questionnaire–Short Form (CTQ-SF) in adolescents from the Peruvian Amazon
Source: Front Psychol. 2026 Oct 8;17:1939936. doi: 10.3389/fpsyg.2026.1939936 (PMC13686878; doi:10.3389/fpsyg.2026.1939936)
Supplement: Supplementary file 1 [file Table_1.DOCX]

**Supplementary Material**

*Spanish adaptation and final version of the Childhood Trauma Questionnaire - Short Form (CTQ-SF) in adolescents from the San Martin Region, Peru*

**Supplementary Table S1. Correspondence between the original CTQ-SF items and their Spanish adapted version, showing the linguistic and cultural modifications applied.**

| **No.** | **Original item (Bernstein et al., 2003)ᵃ** | **Spanish (adapted) version** | **Original dimension** | **Type of modification / justification** |
| --- | --- | --- | --- | --- |
| 1 | *Not enough to eat* | En mi casa hubo veces que no había suficiente comida para alimentarme bien | Physical neglect | Contextual adaptation. The family and food referent was made explicit for greater clarity among adolescents |
| 2 | *Someone to take care of and protect me** | Tuve a alguien que me cuidaba y protegía | Physical neglect | Direct translation. Reverse-scored item; the sense of care/protection was preserved |
| 3 | *Called “stupid,” “lazy,” or “ugly”* | He sido llamado “estupido”, “perezoso” y/o “feo” | Emotional abuse | Lexical adaptation. Insults commonly used in Peruvian Spanish were employed |
| 4 | *Parents too drunk/high to take care* | Mis padres estaban demasiado borrachos o drogados como para cuidarme | Physical neglect | Direct translation. The original meaning of parental incapacity due to substance use was retained |
| 5 | *Someone helped me feel important** | Alguien me ayudó a sentirme importante | Emotional neglect | Direct translation. Reverse-scored item; the sense of personal worth was preserved |
| 6 | *Had to wear dirty clothes* | A veces tenia que usar ropa sucia o en mal estado | Physical neglect | Contextual adaptation. “Or in poor condition” was added to reflect local socioeconomic realities |
| 7 | *Felt loved** | Me sentía amado/a | Emotional neglect | Direct translation. Reverse-scored item; gender-inclusive form was used |
| 8 | *Thought my parents wished I had never been born* | Sentía o pensaba que mis padres deseaban que yo no hubiera nacido | Emotional abuse | Direct translation. The sense of parental rejection was retained |
| 9 | *Hit so hard that I had to see a doctor* | Me golpearon tan fuerte que tuve que ver a un médico | Physical abuse | Direct translation. The severity indicator (medical attention) was retained |
| 10 | *Nothing I wanted to change in my family* | No hay nada que quiera cambiar de mi familia | Minimization/Denial | Direct translation. Validity item; not part of the clinical subscales |
| 11 | *Hit me so hard that it left bruises or marks* | Me golpearon tan fuerte que me dejaron moretones o marcas | Physical abuse | Direct translation. The visible-injury indicator was retained |
| 12 | *Punished with belt, board, cord, or another hard object* | Me castigaron con un cinturón, tabla, cable u otro objeto duro | Physical abuse | Lexical adaptation. Examples of objects were adjusted to those common in the local context |
| 13 | *My family looked out for each other** | Mi familia se cuidaba entre sí | Emotional neglect | Direct translation. Reverse-scored item; the sense of family cohesion was preserved |
| 14 | *My family said hurtful or insulting things to me* | Mi familia me decía cosas hirientes o insultantes | Emotional abuse | Direct translation. The sense of verbal aggression was retained |
| 15 | *Physically abused* | Fui abusado/a físicamente | Physical abuse | Direct translation. Gender-inclusive form was used |
| 16 | *Perfect childhood* | Tuve una infancia perfecta | Minimization/Denial | Direct translation. Validity item; not part of the clinical subscales |
| 17 | *Got hit badly ... noticed by teacher, neighbour, or doctor* | Me golpearon tan fuerte que lo notó un maestro, vecino y/o médico | Physical abuse | Direct translation. The severity indicator observable by third parties was retained |
| 18 | *Someone in my family hated me* | Alguien en mi familia me odiaba | Emotional abuse | Direct translation. The sense of family hostility was retained |
| 19 | *Family felt close to each other** | Mi familia se sentía unida entre sí | Emotional neglect | Direct translation. Reverse-scored item; the sense of family closeness was preserved |
| 20 | *Someone tried to touch me in a sexual way or tried to make me touch them* | Alguien intentó tocarme sexualmente o hacer que yo lo/la tocara | Sexual abuse | Direct translation. The sense of sexual contact was retained |
| 21 | *Threatened to hurt me unless I did something sexual with them* | Alguien me amenazó con hacerme daño si no hacía algo sexual con él/ella | Sexual abuse | Direct translation. The sense of sexual coercion was retained |
| 22 | *The best family in the world* | Tengo la mejor familia del mundo | Minimization/Denial | Direct translation. Validity item; not part of the clinical subscales |
| 23 | *Someone tried to make me do sexual things or watch sexual things* | Alguien me obligó a hacer cosas sexuales o a ver cosas sexuales | Sexual abuse | Direct translation. The sense of sexual imposition was retained |
| 24 | *Someone molested me* | Alguien me tocó de manera inusual o inapropiada | Sexual abuse | Lexical adaptation. “Molested” was paraphrased with understandable, non-stigmatizing wording |
| 25 | *I was emotionally abused* | Fui abusado/a emocionalmente | Emotional abuse | Direct translation. Gender-inclusive form was used |
| 26 | *Someone to take me to the doctor if I needed it** | Alguien me llevó al médico cuando lo necesitaba | Physical neglect | Direct translation. Reverse-scored item; the sense of access to medical care was preserved |
| 27 | *I was sexually abused* | Fui abusado/a sexualmente | Sexual abuse | Direct translation. Gender-inclusive form was used |
| 28 | *My family gave strength and support** | Mi familia me dio fuerza y apoyo | Emotional neglect | Direct translation. Reverse-scored item; the sense of family support was preserved |

*Note. ᵃ Original items are presented in the abbreviated key-word form reported by Bernstein et al. (2003, Table 1), as the full verbatim wording of the instrument is protected by copyright (The Psychological Corporation). An asterisk (*) denotes reverse-scored items. Items 10, 16, and 22 belong to the Minimization/Denial (validity) scale and are not part of the clinical subscales. The Spanish version was obtained through direct translation and back-translation with expert consensus; modifications aimed at semantic equivalence and cultural relevance for Peruvian adolescents while preserving the meaning of the original construct.*

**Supplementary Table S2. Final version of the adapted CTQ-SF (18 items, three factors) and scoring instructions.**

Response format (5-point Likert-type scale):

| **Never true** | **Rarely true** | **Sometimes true** | **Often true** | **Very often true** |
| --- | --- | --- | --- | --- |
| 1 | 2 | 3 | 4 | 5 |

| **No.** | **Item** | **Coding** |
| --- | --- | --- |
| **Factor 1: Perceived family support (7 items)** | | |
| 2 | Tuve a alguien que me cuidaba y protegía | Reverse (R) |
| 5 | Alguien me ayudó a sentirme importante | Reverse (R) |
| 7 | Me sentía amado/a | Reverse (R) |
| 13 | Mi familia se cuidaba entre sí | Reverse (R) |
| 19 | Mi familia se sentía unida entre sí | Reverse (R) |
| 26 | Alguien me llevó al médico cuando lo necesitaba | Reverse (R) |
| 28 | Mi familia me dio fuerza y apoyo | Reverse (R) |
| **Factor 2: Sexual abuse (5 items)** | | |
| 20 | Alguien intentó tocarme sexualmente o hacer que yo lo/la tocara | Direct |
| 21 | Alguien me amenazó con hacerme daño si no hacía algo sexual con él/ella | Direct |
| 23 | Alguien me obligó a hacer cosas sexuales o a ver cosas sexuales | Direct |
| 24 | Alguien me tocó de manera inusual o inapropiada | Direct |
| 27 | Fui abusado/a sexualmente | Direct |
| **Factor 3: Physical-emotional abuse (6 items)** | | |
| 8 | Sentía o pensaba que mis padres deseaban que yo no hubiera nacido | Direct |
| 9 | Me golpearon tan fuerte que tuve que ver a un médico | Direct |
| 11 | Me golpearon tan fuerte que me dejaron moretones o marcas | Direct |
| 12 | Me castigaron con un cinturón, tabla, cable u otro objeto duro | Direct |
| 14 | Mi familia me decía cosas hirientes o insultantes | Direct |
| 17 | Me golpearon tan fuerte que lo notó un maestro, vecino y/o médico | Direct |

*Scoring instructions. Each item is answered on a scale from 1 (never true) to 5 (very often true). The seven items of Factor 1 (Perceived family support) are positively worded and must be reverse-scored (reversed score = 6 - original score) before computing the factor score, so that higher scores consistently indicate greater exposure to maltreatment (in this case, lower perceived family support / greater emotional neglect). Each factor score is obtained by summing its constituent items. Because the structure differs from the original five-factor model, the cut-off scores of the five original CTQ-SF subscales are not applicable to this version.*

*Note. The 10 CTQ-SF items excluded from the final version (1, 3, 4, 6, 10, 15, 16, 18, 22, and 25) were removed due to low factor loadings, content redundancy, or because they belong to the validity scale of the original instrument. The Spanish wording of each item is retained in the original language of administration.*
